# Who Owns Your Data?

**DOI:** 10.1002/acm2.12335

**Published:** 2018-04-24

**Authors:** Michael D Mills

Twenty years ago, when we were exploring the meaning behind starting the first open‐access medical journal, we asked ourselves: Who should own the article? In that era, it was universally expected that all authors must give away their articles to a publisher so the business model will work. The publishers, in turn, sell print advertising, reprints, subscriptions and charge other fees to create and maintain a tidy business. According to Nature, in 2013, the net profit margin for science publishers before tax was estimated at 30%–50%, depending on the publisher and some other circumstances. https://psgsc.wisc.edu/wp-content/uploads/sites/205/2012/09/van-Noorden-2016-.pdf

These profit margins are an order of magnitude greater than most industries. Consider this Table from Forbes, which lists the profit margins of the most profitable industries. (Publishing as a whole is not listed because in the age of electronic media, overall publishing is very low in profitability as opposed to science journal publishing): https://www.forbes.com/sites/sageworks/2015/09/06/these-industries-generate-the-highest-profit-margins/#7293e260310c.

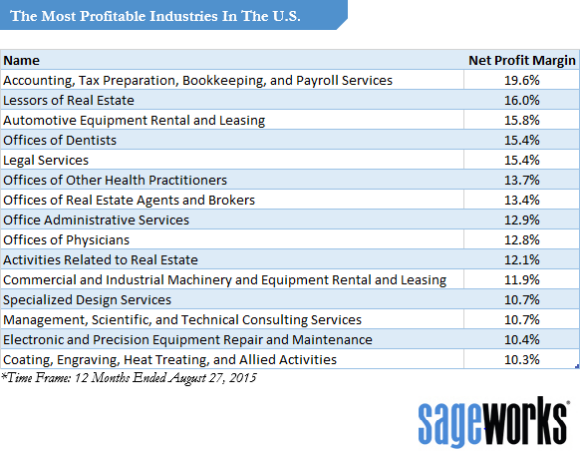

However, today the science and medical journal publishing industry is in transition. Many journals are converting to subscription‐based electronic‐only publication, and this eliminates the revenue from print advertisements. Eventually, these journals may adopt some type of open‐access format as the open‐access journals that exist continue to experience substantial growth. My impression is that the industry is well aware of these sea‐changes and will navigate the transition optimistically and profitably.

But where is the author in all of this? Years ago, we recognized the fundamental problem. The author needs to own the article! Thus, the author controls who can access it and how it can be used in the future. Today, the same dynamic is playing out in the public arena, not with scientific manuscripts, but with all personal and business data. Who owns all of these data?

Data is the new oil, and there are going to be land grab arguments and fights over who owns it. The battles are beginning. The new appliances you are purchasing are becoming part of the so‐called “Internet of Things” (IOT) and will generate data streams. Do you want to own these data? You should be prepared to fight for it. In fact, you may not even be given access to it. Rather, the manufacturers intend to collect the data, analyze it, repackage it and sell it.

Most of those of my generation (baby‐boomers) are not aware that their smartphones come equipped with a GPS which feeds their location and other information back to their mobile carrier and phone manufacturer. The same GPS technology is used in smart tractors to plant a straight row of wheat, determine what fields have the best and worst yields, and calculate when to reseed to get the best return. This is great, but what happens if the large industry companies (read competition!) want that data? Will the farmers be able to sell it? Would they want to? It the future, will the tractors simply send the data directly to the cloud for harvesting by those the manufacturer chooses? Will the data be auctioned? Will the farmer and legitimate owner of the data even be allowed to bid? https://www.wired.com/insights/2014/02/owns-data/

All of us are aware of some recent highly publicized controversies involving the social media giants. Much of this data grabbing feels creepy. It makes a lot of money, but not for the consumer — not for you and me. The mindset to take control of our personal data is parallel to the much simpler problem of determining who owns your scientific article. Today, you have a choice where you publish, and you can retain ownership and publish open‐access if you wish. We need the power to exercise this choice for our personal data as well.

